# A hyperspectral open-source imager (HOSI)

**DOI:** 10.1186/s12915-024-02110-w

**Published:** 2025-01-07

**Authors:** Jolyon Troscianko

**Affiliations:** Centre for Ecology & Conservation, University of Exeter, Penryn, UK

**Keywords:** Hyperspectral imaging, Spectral imaging, Light environment, Artificial light at night, Spectro-radiometry, Ecology, Environmental optical physics

## Abstract

**Background:**

The spatial and spectral properties of the light environment underpin many aspects of animal behaviour, ecology and evolution, and quantifying this information is crucial in fields ranging from optical physics, agriculture/plant sciences, human psychophysics, food science, architecture and materials sciences. The escalating threat of artificial light at night (ALAN) presents unique challenges for measuring the visual impact of light pollution, requiring measurement at low light levels across the human-visible and ultraviolet ranges, across all viewing angles, and often with high within-scene contrast.

**Results:**

Here, I present a hyperspectral open-source imager (HOSI), an innovative and low-cost solution for collecting full-field hyperspectral data. The system uses a Hamamatsu C12880MA micro spectrometer to take single-point measurements, together with a motorised gimbal for spatial control. The hardware uses off-the-shelf components and 3D printed parts, costing around £350 in total. The system can run directly from a computer or smartphone with a graphical user interface, making it highly portable and user-friendly. The HOSI system can take panoramic hyperspectral images that meet the difficult requirements of ALAN research, sensitive to low light around 0.001 cd.m^−2^, across 320–880 nm range with spectral resolution of ~ 9 nm (FWHM) and spatial resolution of ~ 2 cycles per degree. The independent exposure of each pixel also allows for an extremely wide dynamic range that can encompass typical natural and artificially illuminated scenes, with sample night-time scans achieving full-spectrum peak-to-peak dynamic ranges of > 50,000:1.

**Conclusions:**

This system’s adaptability, cost-effectiveness and open-source nature position it as a valuable tool for researchers investigating the complex relationships between light, environment, behaviour, ecology and biodiversity, with further potential uses in many other fields.

## Background

Light carries a wealth of information about the world around us; primarily conveyed in its intensity, spectral composition and angular direction. Taken together, this spatio-spectral information underpins the evolution and function of animal vision, e.g. [1], guides behaviour and ultimately impacts animal evolution via natural or sexual selection [2]. Spatio-spectral information is also used by a range of sensing/measurement systems, including optical physics [3], plant ecology and agriculture [4, 5], food technology, geology/mineral science and astronomy [6]. Quantifying the light field is also critical for accurate modelling of object and scene appearance, linking fields as diverse as human psychophysics, visual ergonomics, lighting design and architecture and digital creative industries [7, 8].

The light environment is fundamental to all aspects of visual ecology, from behaviour to evolution, and can even drive speciation [9, 10]. Natural scenes vary substantially in their spatio-spectral properties [11], largely driven by the type of illuminant (such as sunlight, moonlight, starlight, bioluminescence or artificial light), and also how the light propagates through the environment. Factors such as scattering (e.g. Rayleigh scattering or oceanographic backscattering), cloud/fog/forest canopy/algal absorption and Snell’s window determine the intensity, spectral composition and spatial structure of the light environment [1, 12–15]. For example, a cloudy/foggy day will result in isotropic illumination (spatially uniform illumination), reducing shadows and contrast. Direct light (such as sunshine) will result in interactions between the habitat’s 3D structure and lighting angle to create strong shadows likely to increase the scene’s visual complexity and make visual search more difficult [16]. The light’s directional properties will also affect critical anti-predator defences such as countershading, which is dependent on the intensity of an animal’s self-shadows [17].

Artificial light at night (ALAN) is increasingly recognised as a global threat to biodiversity [18–20], and over the past 18 years, its intensity has more than quadrupled [21]. Artificial light spectra differ substantially from natural sources, and the detrimental impact of these emissions varies among animals and plants that also have enormous variation in spectral sensitivity [19, 22, 23]. Existing data on the global extent of ALAN are largely limited to satellite-based data that measure upwelling light on cloud-free nights and so are unable to account for atmospheric effects (such as skyglow; [21, 24]) and horizontally propagated light. These aspects of the light environment alter its directional structure, which in turn can affect animal anti-predation responses [25]. Quantifying the light environment’s spatial and spectral properties is therefore of particular importance for ALAN research.

A number of existing methods have been developed for quantifying spatio-spectral information. No clear consensus exists on the distinction between multispectral and hyperspectral imaging systems [26]; however, in the context of vision research ‘hyperspectral’ is generally reserved for systems that operate at substantially higher spectral resolution than tristimulus RGB devices designed for human colour vision (with spectral resolution limited by typical opsin responses) and/or systems that measure a wider range of wavelengths than human vision [27]. Multispectral techniques such as wide-angle photography can be used to photograph whole scenes, with calibration allowing for conversion to absolute radiance [28]. Further recent work has used similar methods to model non-human low-light visual dynamics to ALAN and sky glow in particular [24]. This method enables rapid data collection using affordable equipment, with high spatial resolution and moderately high dynamic range. The dynamic range limits of a camera sensor can be partially overcome by using exposure bracketing techniques; however, this cannot overcome the contrast limits of the optics; lenses have internal reflections and contrast limits characterised by a modulation transfer function [29] result in under-estimates of true scene dynamic ranges, particularly where point light sources cause glare. Photography-based methods also have limited spectral resolution, typically just three bands similar to human cone functions.

Multispectral and hyperspectral camera systems that are sensitive to a wider spectral range (e.g. ultraviolet (UV)-visible range) use pass filters as a compromise between spatial and spectral resolution under natural lighting for many tasks [11, 30]. However, wide-angle lenses are not available in the UV–visible range because few grades of optical glass transmit UV, and these have similar refractive indices and Abbe numbers (so cannot easily bend the light sufficiently), while anti-reflective coating options are limited. Moreover, the spectral resolution of typical multispectral imagers is not sufficient for ALAN work that often involves complex emission spectra (resulting in metamerism in low-spectral resolution systems [31]). These camera systems also require moderately complex calibration to convert pixel values to absolute radiance.

Hyperspectral cameras can be used to image reflective spheres in order to build up whole-scene radiance with higher spectral resolution than that offered by camera-based systems (as used by [7]). Hyperspectral cameras typically either separate the spectrum onto the sensor and scan mechanically through space for the second spatial axis (push broom) or scan through the spectrum (effectively a multispectral camera with more bands) to build up a hyperspectral data-cube. Each has its own costs and benefits. These systems can provide moderately high spectral and spatial resolution; however, there are a number of drawbacks. For example, spatial scanning can be difficult to calibrate because sensitivity varies across the sensor, while spectral scanning can have limited spectral resolution [32]. Importantly, the dynamic range of these hyperspectral cameras is comparatively low because the optics and exposure control must now operate over an extra spatial or spectral dimension simultaneously. Absolute sensitivity is also typically low, making them poorly suited to low-light situations (such as ALAN research). Wide angle or whole-scene capture is also complex as it requires stitching together multiple scans from different angles to build up a whole scene on top of the above calibrations required to convert to absolute radiance. Hyperspectral cameras are also comparatively expensive (typically > £20 k for human-visible range or considerably more for UV–visible systems) and far beyond the resources of many researchers. Whisk broom hyperspectral imagers are motorised (gimbal-mounted) spectroradiometers that build up the image spatially pixel-by-pixel, such as the open-source system developed by Nevala and Baden [27]. The ASTMON systems also uses this method for quantifying night-time lighting for astronomy [6]. Advantages of this system are that its optics can be shielded from the wider scene (limiting glare and chromatic aberrations), while the main disadvantage is much slower scanning over the two spatial axes, and more moving parts. Nevertheless, such systems are not typically affordable, accessible or easily adapted to measuring high dynamic range light environments. I therefore developed a low-cost, high-sensitivity imaging system capable of producing spectral radiance or relative reflectance images of whole-scenes across wavelengths relevant to any visual system.

## Results

Here and in the “Methods” section, I describe the system, detail the calibration process and present real-world demonstrations. All code, calibration data and sample scans are provided as supplementary information with this submission and as a Zenodo repository [33]; however, see the GitHub project for the latest updates: https://github.com/troscianko/HOSI/. In addition, a series of walkthrough videos are provided to demonstrate assembly, firmware upload, calibration and GUI software functionality; links are provided below.

HOSI radiance and reflectance accuracy data are illustrated in Figs. 3c and d, which measured six artist’s pastels (chosen due to their variable UV reflectance), plus a 99% Spectralon standard. The scene was arranged with diffuse illumination from above (using a stable xenon arc lamp, see the “Methods” section) and measured at roughly 45° with the HOSI and the Jeti Specbos. Radiance and reflectance data were extracted from three pixels in the hyperspectral image using the HOSI GUI software. Mean absolute error in radiance accuracy from 350 to 750 nm was 1.99% (relative to the Jeti’s peak radiance measurement), and mean absolute error in reflectance was 2.5%. These values are likely conservative due to a number of additional sources of error from this test, including (i) instability in illuminant spectral emissions over time, (ii) differences in measurement angle and location (the pastels were sanded, but are not perfect Lambertian reflectors) and (iii) error/differences in the acceptance angle of the Jeti Specbos.

Spatial resolution tests were performed by scanning a square-wave red-blue grating of fixed angular width on an liquid crystal display (LCD) display screen from a distance of 435 mm. The HOSI was set to measure a horizontal transect at its highest spatial resolution of 1 stepper-motor step per measurement (providing a theoretical maximum of 0.25° per cycle). The grating width (defined as a full cycle of red-to-blue) was adjusted on the screen to match specific degrees-per-cycle. Contrast was measured from HOSI’s image output; the highest amplitude cycle in each image was measured; these values were linearised, and contrast was calculated as the amplitude relative to the peak contrast at a grating width of 4°. The contrast function is shown in Fig. 5; spatial contrast fell to 50% of the maximum at ~ 0.71° per cycle. This implies that improving the optics (rather than mechanics) would be required to improve spatial resolution.

Scan speed testing shows a maximum scan speed of ~ 11.8 scans/second for bright scenes (~ 2000 cd.m^−2^). This reduces to 3.6 scans/second for integration times of 3200 microseconds (~ 200 cd.m^−2^). Low-light scenes (< 1 cd.m^−2^) can decrease scan speed substantially. For example, exposure times of 1 s reduce the speed to 0.46 scans/second. In practice, a daytime woodland scene (86 × 23 scan) took around 20 min, while the nighttime docks scene (61 × 16 scan, Fig. 2b) took roughly 40 min; both scans are included in the sample data.

## Discussion

The hyperspectral open-source imager (HOSI) presented in this study addresses the growing need for affordable and versatile tools for quantifying spatio-spectral properties of light environments. In particular, it offers high spectral and spatial resolution, high sensitivity and a far larger dynamic range than existing ultraviolet to near infrared systems (320–880 nm). Commercially available UV–visible range photographic/hyperspectral imaging lenses are expensive (typically around $10,000), suffer from internal reflections that reduce contrast and offer limited focal length ranges that make whole-scene capture difficult. Spatially scanning (push broom) hyperspectral imagers also suffer from low contrast due to the need to expose the sensor across space and spectrum simultaneously. HOSI overcomes these problems by combining simple, affordable optics that are focused on only one part of the scene at a time, thereby shielding the sensor from glare. Dynamic range is further enhanced by exposing each pixel independently [27]. Figure 2 shows a night-time scene measured with HOSI, demonstrating how individual bulb technologies can be identified, while also highlighting the large dynamic range present (with the highest spectral peak over 50,000 times higher than the lowest spectral peak in the scene). HOSI also offers benefits when compared to the comparable whisk broom system developed by Nevala and Baden [27], including approximately three times faster maximum scan rates, approximately four times higher spatial resolution, far greater low-light sensitivity, the ability to scan over more than a hemisphere, higher dynamic range (because each pixel is independently exposed) and considerably cheaper components. Moreover, the system is small and portable, capable of being operated with a smart phone, and has comparatively straightforward calibration compared to existing solutions, with output data that are largely pre-processed (including radiance spectra, reflectance spectra and relative cone-catch images).

While HOSI offers significant advantages, a notable limitation is the time it takes to scan high-resolution images, particularly under low-light. As such the system is not suited to instantaneous imaging of scenes with fast-changing illumination or moving objects. However, high spatial resolution is not always required for quantifying the light environment, and in many cases movement or spatial changes may be statistically averaged in large datasets. Moreover, the system’s flexibility allows researchers to adjust elevation and azimuth resolutions independently based on their specific needs, balancing time constraints with spatial requirements. For example, Nilsson and Smolka [28] use whole-scene photographic techniques with high spatial resolution, but the data are subsequently converted to vertical transects that a HOSI could readily replicate through measuring transects from the outset. Alternatively, when high spatial resolutions are required, HOSI can be used in the lab with a stable full-spectrum light source where capture time is not limiting.

## Conclusions

The HOSI’s adaptability and open-source nature contribute to its potential for environmental sensing and light environment quantification in a wide range of scientific disciplines. Furthermore, its affordability and ease of automation makes it well placed for future adaptation to high-risk environments (aerial or underwater sensing [15]) or at increased scope by deploying multiple systems. The project also paves the way for future innovations in spatio-spectral data measurement systems that require co-ordination of spectrometry with actuators, such as automated goniometry for quantifying iridescence [34], or automated sensing systems in medicine or food testing.

## Methods

### Hardware

**Fig. 1:**
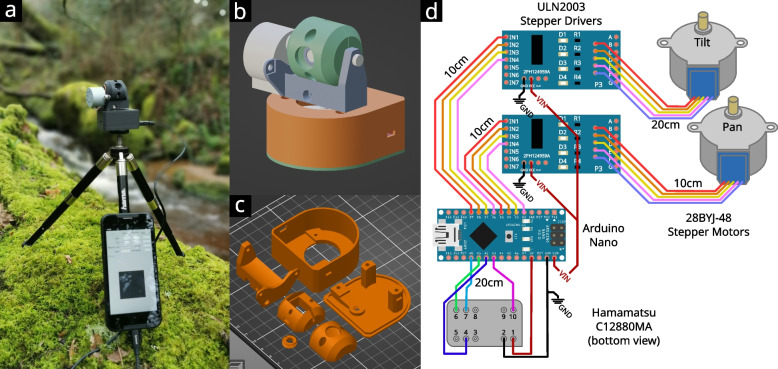
3D parts and wiring. **a** Photograph of a HOSI unit in the field, running from a smartphone. **b** 3D assembly. **c** Parts arranged for 3D printing. **d** Wiring diagram and required wire lengths. Ground pin on electronic board (GND) (ground) is the shared negative voltage; positive voltage input pin on electronic board (VIN) is the 5v output from the Arduino that is delivered via the USB cable. Note that the spectrometer should be connected to the Arduino’s regulated 5v output (as shown), not VIN that has a slightly lower voltage due to a protective diode in the Arduino

HOSI was developed from open source spectroradiometer (OSpRad), an open-source spectroradiometer designed for low-light spectral radiance and irradiance measurements [31]. Both systems are built around the Hamamatsu C12880MA micro spectrometer, which has comparatively high sensitivity (down to 0.001 cd.m^−2^), a high spectral resolution with 288 photosites (typical full width at half maximum (FWHM) of 9 nm, and maximum FWHM of 1 5 nm) and a spectral range of ~ 320–850 nm that encompasses effectively all animal photo-transduction-based visual systems and the photosynthetically active radiation (PAR)/near-infrared (IR) ranges required for plant-based measurements. The component is low-cost (approximately £220 from Hamamatsu UK) and is typically used in medical equipment but can be interfaced through a customised open-source microcontroller. For further details on how the microcontroller interfaces with the C12880MA, see [31] and the HOSI’s C + + Arduino code. Parts were printed in matt black PLA (Polylactic acid, SUNLU Matte PLA 1.75 mm Printer Filament, Amazon UK, ~ £18, Fig. 1c). It is essential that the printed plastic does not transmit near-infrared light (e.g. avoid black PET, polyethylene terephthalate). See Fig. 1 for examples.

**Fig. 2:**
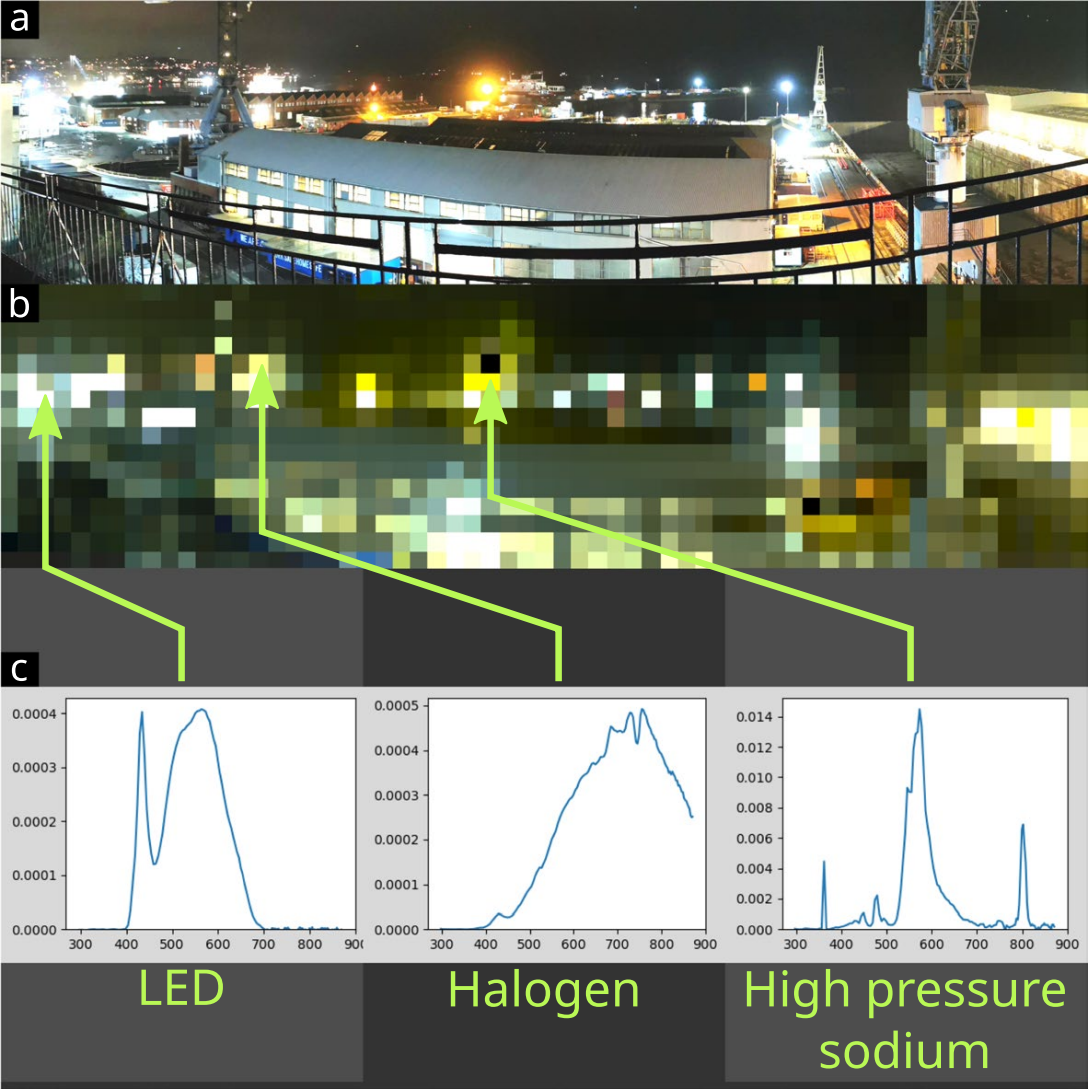
Example hyperspectral scan of Falmouth (UK) docks at night. **a** Panoramic photograph of the scene taken with a Huawei P30. **b** HOSI’s hyperspectral RGB preview image. **c** Radiance plots created by the HOSI GUI at chosen pixels (*x*-axis: wavelength [nm]; *y*-axis: linear radiance *Le* [W.sr^−1^.m^−2^.nm^−1^]). These spectra illustrate how individual light sources can be identified and compared. The highest spectral peak in the brightest pixel of this hyperspectral image is approximately 50,000 times higher than the peak in the darkest pixel. The full dynamic range (highest peak to lowest trough) is considerably greater and can be maximised further with longer exposure times to reduce noise

Focusing is achieved using a biconvex fused silica lens with 10 mm focal length and 6 mm diameter (LB4280; Thorlabs Ltd. ~ £75). This is an off-the-shelf lens that provides sufficient spatial detail for most imaging and light environment measurement tasks. The lens is placed into a 3D-printed threaded holder that can be screwed into the HOSI housing for manual focusing from infinity to roughly 30 mm. A 3D printed tool allows for easy focusing, and a user can make marks on this tool to denote the positions of different focal depths. This system is well suited to low-light application, but will saturate under strong daylight light intensities, so I provide 3D parts for a light reducer aperture for daytime use (note that the spectral sensitivity calibration below must be repeated if a light reducer is used) (Fig. 2).

Hyperspectral images are acquired by using a 3D printed gimbal that pans and tilts the spectroradiometer with stepper motors. 28BYJ-48 stepper motors were selected for their high angular resolution of 2048 steps per revolution, an ability to run from a USB (5v) source, their availability and low cost. The spectrometer and stepper motors are controlled by an Arduino Nano microcontroller; this in turn interfaces through serial connection with a computer or smart phone. The housing uses 3D printed parts and can be assembled with basic soldering skills (Fig. 1d).

### Calibration

#### Linearisation

**Fig. 3:**
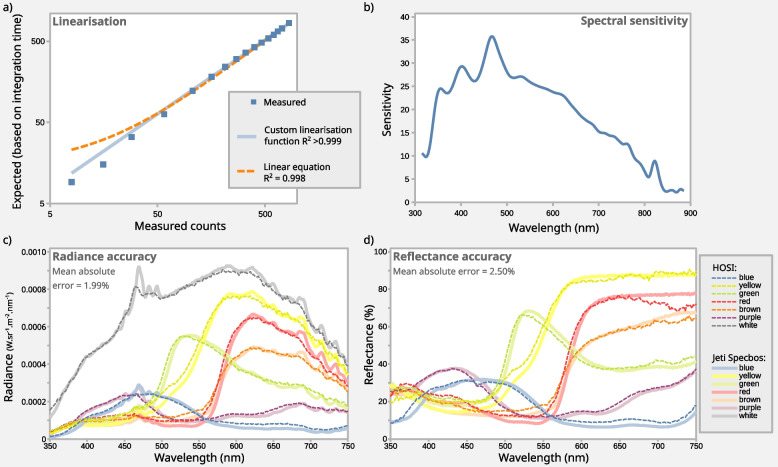
Plots showing calibration and verification data. **a** shows output from the automated linearisation protocol that uses integration time to calculate the sensor response. Log–log axes highlight the accuracy of the fit at low values and how a linear fit would deviate from this near zero. **b** shows spectral sensitivity as calculated by the spectral calibration script, comparing HOSI measurements to NIST-traceable calibration data. Plots **c** and **d** show the radiance and reflectance data from the HOSI of six pastels illuminated by an Osram XBO lamp, compared to Jeti Specbos calibration data. Quoted error values are in the range 350–750 nm, outside of which error increases in both systems due to low lamp emissions

The C12880MA chip has a good linear response to radiance across most of its range (*R*^2^ values typically ~ 0.996–0.998); however, low count values (below roughly 1% of maximum) over-estimate radiance relative to the middle and upper ranges (see Fig. 3a). To streamline the linearisation process, I developed a Python script (file: Calculate_HOSI_linearisation_curve_0.6.py) that automates the process by taking a series of exposures with known integration times ranging from 0.005 to 1.5 times the auto-exposed integration time. These integration times can then be compared to sensor count values to develop a linearisation function. To use the script, the HOSI should be placed in front of a surface that is illuminated with a source that will remain stable in spectral output and intensity for roughly one minute. For most reliable results, this should be performed in a dark room to eliminate unwanted light ingress during the dark measurement. Almost any stable non-flickering source can be used in principle, such as conventional non-flickering LED room lighting or a display screen. Ideally, the intensity should use upper integration times of around 200,000–800,000 microseconds to maximise the dynamic range (adjust the brightness of the source to achieve these exposure values). The script removes any saturated (over-exposed) values, a Gaussian smoothing function (*σ* = 3) is applied to the spectral recordings to reduce noise, dark count values are subtracted from light counts and the wavelength bin with the highest (peak) response is selected for further calculations. Values at this peak wavelength are then compared between known exposure times, comparing observed and expected responses (based on known and precisely controlled integration times). The script then uses least squares regression to fit the results to the linearisation equation:

$$\text{Linear counts}, {c}_{t}={e}^{\text{ln}\left({r}_{t}-{d}_{t}\right)a+b}$$

where *r* is the raw count value at integration time *t*, *d* is the corresponding dark count value at the same integration time and *a* and *b* are the linearisation coefficients. The script outputs the coefficients together with *R*^2^ values and log–log axis plot. This linearisation modelling typically increases *R*^2^ values to > 0.999, with performance at low-values notably improved (fit shown in Fig. 3a).

#### Spectral sensitivity

A custom-assembled stable xenon lamp was used to provide continuous illumination in the UV–visible range (Osram XBO R 100 W/45 bulb [~ $435], driven by a USHIO SMARTARC™ HBX-76 igniter and ballast [~ $500]), both sourced from LabGear Inc. Note that sunlight may also be used as a broadband light source for calibration, although it typically lacks sufficient intensity in UV below ~ 350 nm. The lamp was fitted to a stainless steel hemispherical reflector (made from an IKEA BLANDA bowl £5). Near infrared emissions were reduced by fitting a SCHOTT KG1 filter (3 mm thick, 50 mm diameter, £68, Edmund Optics), mounted 50 mm from the bulb in an aluminium housing. The XBO lamp provides a highly focused beam of light that is not suitable for diffuse illumination. To diffuse the light source, an 80-mm-diameter disk of 1-mm-thick virgin polytetrafluoroethylene (PTFE) sheet (Bay Plastics, www.plasticstockist.com) was fitted roughly 100 mm from the bulb. A second, larger diffuser disk 300-mm diameter made from 0.5-mm-thick virgin PTFE was then used for further diffusion mounted roughly 150 mm from the bulb. A centrifugal fan at the base of the lamp provides cooling to the bulb and diffusers (the ballast provides a 12v output for this purpose). The resulting lamp is comparatively affordable and provides unfocused, roughly cosine-shaped throw that is well suited to typical calibrated photography/hyperspectral imaging (as opposed to commercially available lamps that are typically highly focused). However, care must be taken to house and use the system in a safe way given its ionising radiation emissions, high voltage spikes at ignition, heat generation and potential for explosion of the high-pressure XBO lamp. Spectral sensitivity calibration was achieved by taking a hyperspectral image of a Spectralon 99% reflectance (Labsphere) illuminated by the stable xenon source (above) and comparing this to a calibrated spectral radiance measurement. The illuminant was directly above the standard (90° from the surface), while measurements were taken from approximately 45° using a HOSI and a National Institute of Standards and Technology (NIST)-tracable Jeti Specbos UV-1211 spectroradiometer. Raw count data *r*_*t*_ were then extracted from the hyperspectral image file (averaged across the 35 pixels that recorded the white standard), together with count data of the dark measurements *d*_*t*_ taken before and after the white standard, matching the same integration time. Average dark counts are then subtracted from average light count data at each wavelength (i.e. *r*_*t*_*-d*_*t*_). The supplied Python script (file: Calculate_HOSI_spectral_sensitivity_0.3.py) can then be used to calculate the spectral sensitivity curve from these count data and calibrated spectrum, together with the integration time (in microseconds) and the linearisation coefficients (measured above, see code for specific details).

Calibration data must be saved to the calibration_data.txt file in the same directory as the GUI’s Python code (see the walkthrough videos for further details). This file specifies the photosite wavelength calculation coefficients (provided with each Hamamatsu C12880MA chip by the manufacturer), linearisation model coefficients and spectral sensitivity functions for each unit. Each HOSI unit can have its own unit number that is specified when uploading the firmware, and the calibration_data.txt file allows for multiple units to be added (so labs can use multiple units across smartphones/computers without risk of using the wrong calibration data). All testing was performed at standard room temperatures of around 19–20 °C. Sensor performance may vary at temperatures well outside this range, and users should calibrate and test their units under typical use scenarios.

### Firmware

The Arduino Nano microcontroller uses custom written code (‘firmware’, file: Arduino_HOSI_1.05.ino, written in Arduino C + +) that was developed from the OSpRad project as above (Troscianko, 2022); however, it has a number of major changes to optimise the system for speed while maintaining high-dynamic range and providing gimbal positional control. This firmware is uploaded to the Nano using Arduino IDE software (see walkthrough video for further details).

#### Dark measurement

The spectrometer uses a complementary metal–oxide–semiconductor (CMOS)sensor, which converts absorbed light at photocells into a potential difference (higher voltage for more light received), an analogue-to-digital-converter on the Arduino Nano then converts this to a digital count. However, each photocell also has a ‘dark current’ associated with the black point, and this varies with factors such as supply voltage and sensor temperature, so it must be measured regularly. HOSI automatically makes regular (and user customisable) dark measurements by rotating the spectrometer down into the gimbal housing at the end of a panning scan, to a position designed to block light. These dark measurements are stored in the raw output for subsequent calculation of absolute spectral radiance.

#### Exposure control

Exposures are calculated independently at each gimbal position, with integration times starting from the minimum of 500 microseconds, and increasing in octaves until an exposure is saturated in any wavelength or the user-specified maximum integration time is reached (default of 2 s, maximum 30 s). The firmware then sends the previous (non-saturated) exposure to the app (below) and proceeds to the next gimbal position. The theoretical dynamic range of the system within a single hyperspectral image is therefore approximately 1:60,000 based on integration times alone and considerably higher when accounting the dynamic range of the sensor itself (theoretically around 1:540,000,000).

#### Scanning behaviour

The user can specify the start point, stop point and resolution (steps per measurement) for the pan and tilt axes independently. Panning allows measurements across 360° (azimuth) and tilt from approximately − 20 to 90° (elevation). The stepper motor gears introduce a small amount of play that the firmware overcomes by overshooting and returning to position when starting a new panning scan.

#### Projection

HOSI uses a Mercator projection to link each pixel’s azimuth (pan) and elevation (tilt) to 2D image *x* and *y* coordinates respectively. This would result in higher resolution measurements near the poles (high elevation measurements), and as such the firmware progressively skips redundant pixel scans near the poles, interpolating measurements for efficient whole-scene scans (see the woodland scene example included in the sample scans).

#### Spectral compression

Spectral data can be compressed by selecting an averaging value that combines neighbouring photosite count data over a user-specified window (boxcar) size. For example, a value of 2 reduces the number of spectral data by half with minimal reduction in effective spectral resolution (given the C12880MA’s ~ 9n m FWHM resolution). Window sizes greater than 2 will result in loss of spectral resolution.

### Graphical user interface (GUI) application

**Fig. 4:**
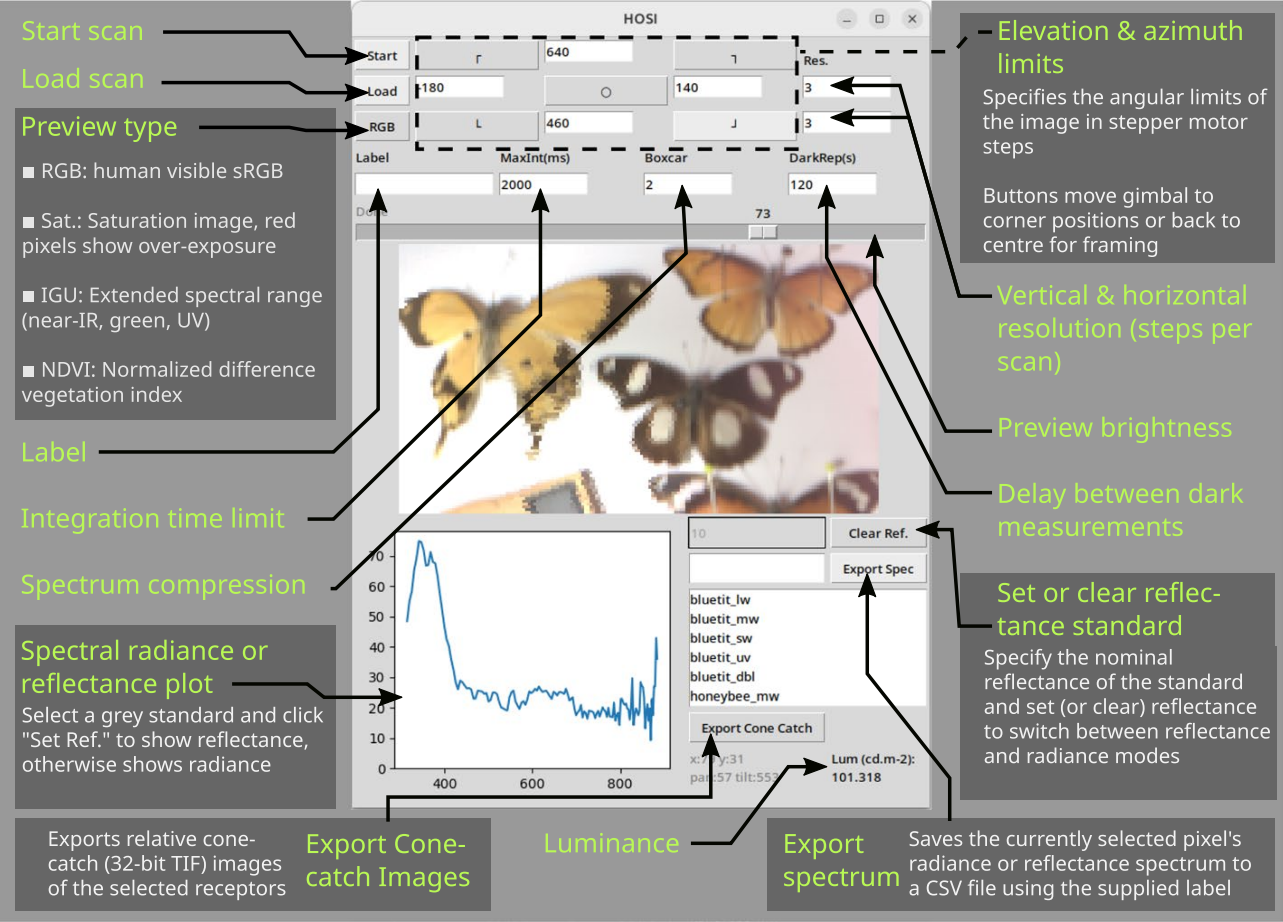
Graphical user interface (GUI) layout, showing the available functions and a scan of butterflies. The spectrum shows a selection from a patch of wing that appears black to human vision but has a UV peak visible to many other animals. This scene was illuminated by an Exo-Terra Sunray lamp

**Fig. 5:**
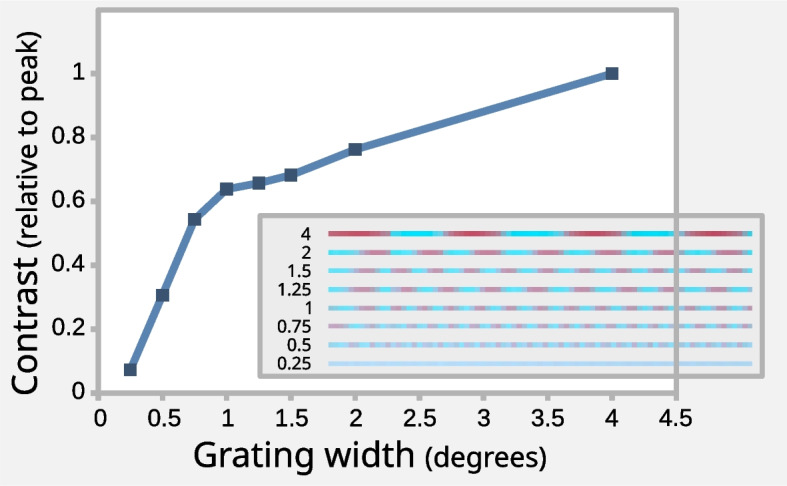
Spatial resolution testing, showing the contrast (relative to 4-degree peak) measured by HOSI for different grating widths, showing a 50% contrast loss below ~ 0.71° per cycle. Inset panel shows the scan transects at each grating width

The HOSI unit receives both power and communication via its USB connection with a computer or smartphone, making the system highly portable and flexible. The graphical user interface application (app, file: HOSI_GUI_0.1.51.py) was written in Python 3.10.6 and provides straightforward access to control a HOSI unit and save the output data to the computer/smartphone (see Fig. 4). The code can run on computers with Python or on Android smart phones using the free Pydroid app. The app uses the calibration data (above) to calculate radiance for each pixel (*Le* [W.sr^−1^.m^−2^.nm^−1^]), which is saved in the output file along with raw data, an sRGB image of the scan, a user-specified label and timestamp (Fig. 5).

The app supports multiple preview formats, including RGB (conventional sRGB image), an extended spectral range image that reassigns the R, G, and B channels to light from other bands (R = near infrared, G = CIE Y, and B = UV), a saturation preview mode that highlights any pixels where saturation has occurred through over-exposure and a normalized difference vegetation index (NDVI) image for vegetation.

Each pixel’s spectral radiance (*Le W.s*^−*1*^*.m*^−*2*^*.nm*^−*1*^) can be plotted by tapping/clicking on it in the preview. Output can also be converted to relative reflectance by selecting a pixel containing a grey or white standard and clicking ‘Set Ref.’. Once set, pixel spectrum plots now show relative reflectance (% relative to the standard), and the preview image has the white-balance set. The ‘Export Spec.’ button saves the current spectrum (either radiance or reflectance depending on whether a standard has been selected) to a.CSV file alongside the scan file. The ‘Export Cone-Catch’ outputs quantal cone-catch images (as 32-bit TIFs) of the selected channels to the working directory. Custom receptor data can be added to the ‘sensitivity_data.csv’ file.

## Data Availability

All data are included as supplementary files with this submission. All code, 3D models, calibration data, and sample scans are available at the project’s GitHub page: https://github.com/troscianko/HOSI/, released under a GNU General Public License v3.0.

## Abbreviations

ALAN: Artificial light at night
HOSI: Hyperspectral open-source imager
FWHM: Full width at half maximum
cd.m^−2^: Candelas per metre squared
UV: Ultraviolet
IR: Infrared
VIN: Positive voltage input pin on electronic board
GND: Ground pin on electronic board
3D: Three-dimensional
UK: United Kingdom
W.sr^−1^.m^−2^.nm^−1^: Watts per steradian per metre squared per nanometre
LCD: Liquid crystal display
OSpRad: Open source spectroradiometer
PAR: Photosynthetically active radiation
PLA: Polylactic acid (plastic)
PET: Polyethylene terephthalate (plastic)
PTFE: Polytetrafluoroethylene (plastic)
NIST: National Institute of Standards and Technology
CMOS: Complementary metal–oxide–semiconductor
GUI: Graphical user interface
RGB: Red, green, blue
sRGB: A standardised RGB colour space
CIE: International Commission on Illumination
NDVI: Normalized difference vegetation index
CSV: Comma-separated values
TIF: Tagged image file format
